# Effect of childhood pneumococcal vaccination and beta-lactam antibiotic use on the incidence of invasive pneumococcal disease in the adult population

**DOI:** 10.1007/s10096-021-04196-4

**Published:** 2021-02-27

**Authors:** Abelardo Fernández Chávez, Luis García Comas, Juan Carlos Sanz Moreno, Rafael Cantón Moreno, Octavio Corral Pazos de Provens, Jesús María Aranaz Andrés

**Affiliations:** 1 Preventive Medicine and Public Health, Hospital Universitario Ramón y Cajal, IRYCIS, Madrid, Spain; 2 Epidemiology Service of Health Department of Community of Madrid, Madrid, Spain; 3 Regional Public Health Laboratory of the Community of Madrid, Madrid, Spain; 4 Microbiology Service, Hospital Universitario Ramón y Cajal, IRYCIS, Madrid, Spain; 5 Microbiology Service, Faculty of Health of UNIR, Madrid, Spain; 6 Preventive Medicine and Public Health, Hospital Universitario Ramón y Cajal, IRYCIS, CIBER of Epidemiology and Public Health (CIBERESP), Madrid, Spain

**Keywords:** Invasive pneumococcal disease, Pneumococcal conjugate vaccine, Reduced antibiotic sensitivity, Beta-lactam antibiotic

## Abstract

Describe the incidence of invasive pneumococcal disease (IPD) in serotypes with reduced antibiotic sensitivity to penicillin (RAS-Pen) in adults over 59 years of age and its association with childhood anti-pneumococcal vaccination coverage (CVC) and community consumption of beta-lactam. We selected IPD cases in adults over 59 years of age reported in the Community of Madrid between 2007 and 2016. We estimated the incidence of cases caused by serotypes included in the 13-valent pneumococcal conjugate vaccine (PCV13), those not included (non-PCV13) and the six serotypes additional to the 7-valent (PCV13-no7). We compared the incidences of serotypes from the pre-vaccine period (2007–2009) and the vaccine period (2011–2016) by analysing the incidence trend (JointPoint Trend Analysis) and its association with the CVC and community consumption of beta-lactam (Poisson model). We identified 1936 cases of IPD, 29.2% (*n* = 565) in serotypes with RAS-Pen. The incidence decreased for PCV13 cases (annual percentage of change, APC: -12.2, *p* < 0.05) and increased for non-PCV13 (APC: 15.4, *p* < 0.05). The incidence of IPD due to non-PCV13 was associated with community beta-lactam consumption (IRR 1.156; CI95% 1.025–1.304) and that of cases of PCV13-no7 with CVC (IRR 0.574; 95% CI95% 0.413–0.797). The non-PCV13 strains that increased the most at the end of the period were 6C, 11A and 15A. The incidence of IPD due to PCV13 with RAS-Pen at > 59 years was decreasing and was associated with CVC. The incidence of cases due to non-PCV13 was increasing and was associated with community consumption of beta-lactam.

## Introduction

In recent decades, a significant worldwide increase in antimicrobial resistance to *Streptococcus pneumoniae* (SP) has been observed [1]. There appears to be numerous factors involved in the evolution of this resistance. Among these, age, vaccination coverage and community antibiotic consumption appear to be the most relevant population-based factors [2].

Adults aged over 59 years are at higher risk of invasive pneumococcal disease (IPD) caused by serotypes with lower antibiotic sensitivity (RAS) because of greater comorbidity, predisposition to infection and high antibiotic consumption [3]. The likelihood of contracting pneumococcal pneumonia or IPD is four times higher in persons over 60 years of age compared to those aged 18 to 49 years [4].

In 2000, the USA became the first country to introduce the first pneumococcal conjugate vaccine in the childhood immunisation schedule that included the seven serotypes (VCN7) that were responsible for most IPD at that time. The high childhood vaccine coverage achieved reduced the incidence of IPD cases among the target cohorts and also the general population [5]. The latter is due to the indirect effect of the high vaccine coverage of children under 2 years, who are the main reservoirs and transmitters of SP to the rest of the population [6].

The lower incidence of IPD caused by the serotypes in the VCN7 contrasted with the higher incidence of cases caused by serotypes not included in the vaccine [7–9], especially 19A, which is particularly resistant to antibiotics. This led to the development of the 13-valent vaccine (VCN13), which included the six most frequent non-vaccine serotypes excluded from its predecessor. As with VCN7, we may now be faced with the emergence of RAS to serotypes not included in VCN13.

VCN7 was included in the Community of Madrid’s child vaccination schedule in 2006, and IPD was added to the list of mandatory notifiable diseases (EDO) shortly thereafter. In June 2010, the VCN7 (serotypes: 4, 6B, 9 V, 14, 18C, 19F, 23F) was replaced by VCN13 (additional serotypes: 1, 5, 7F, 3, 6A, 19A), which was excluded from the child immunisation programme in 2012 and reinstated in May 2015. During this exclusion period, it continued to be administered with private financing [10].

Community antibiotic consumption is also associated with a higher incidence of cases of IPD caused by serotypes with RAS, particularly to penicillin (RAS-Pen) [11]. Some studies show an increase in cases of serotypes with RAS-Pen in areas with higher antibiotic consumption [12, 13]. The Community of Madrid (CM) has one of the highest community consumption of beta-lactam antibiotics in Europe, which are considered first-line antibiotics in the treatment of infectious respiratory diseases [6, 8].

The impact of the above factors on the incidence and distribution of resistant serotypes therefore merits analysis.

This study aims to describe the evolution of the incidence of RAS-Pen by IPD-serotype in the adult population over 59 years of age in the 2007–2016 period and to analyse its association with childhood pneumococcal vaccine coverage and community beta-lactam antibiotic consumption.

## Methods

### Study type

This study is a retrospective descriptive, analytical study**.**

### Selection criteria

We selected cases of IPD in adults over 59 years of age, reported to the System of the Epidemiological Surveillance Network of the Community of Madrid (EDO) between 2007 and 2016. The definition of IPD considers microbiological criteria alone: isolates, DNA detection or detection of SP antigen in samples from normally sterile sites. The definition of RAS to penicillin considers a minimum inhibitory concentration of penicillin greater than 0.06 mg/L in the antibiogram. This definition includes resistant isolates and those presenting intermediate penicillin sensitivity.

### Data sources and variables

The following data sources and variables were consulted and analysed:

- EDO system: data such as date of birth, sex, date of onset of symptoms and microbiological results (serotype and antibiotic sensitivity) collected using a structured form. Serotypes were identified using the Quellung reaction, and antibiotic sensitivity was assessed using the criteria established by *the* European Committee on Antimicrobial Susceptibility Testing (EUCAST) [14].
- Vaccination information system: date of birth, sex, date of vaccination and type of vaccine prescribed.
- Information and analysis system of the pharmaceutical service: community consumption of beta-lactam antibiotics, dispensed by prescription in pharmacy offices. Consumption was expressed as a defined daily dose number (DDD), which is a standardised measure formulated by the WHO (classification system with defined daily doses (ATC/DDD) [15]. For beta-lactam antibiotics, the DDD for the entire Community of Madrid were added together.
- Continuous census of the Institute of Statistics of the CM: population by age, sex and year of study recorded in the CM.

### Indicators

- Cumulative incidence (CMI) of cases of the groups of RAS and penicillin-sensitive serotypes per 100,000 people. The denominator used was the annual number of adults over 59 years residing in the CM.
- Childhood vaccination coverage (CVC): We estimated the annual first-vaccinated in the 2-year-old cohort, this being the age at which first vaccination with two or three doses was theoretically received under the current vaccination schedule. First-vaccinated was defined as children vaccinated without the booster dose. Vaccination coverage was categorised into three levels for multivariate analysis (< 85%, 86–90% and > 90%). Vaccination coverage was calculated for VCN13 (VC13) and for the sum of the two vaccines (VC7 + 13).
- Daily defined dose per 1000 persons per day (DHD) of adults over 59 years of age: calculated for each study year. The following formula was used: DHD = (DDD*1000) / population*365 [15].

### Analysis

Five groups of IPD cases were considered according to the causative serotypes: (1) any serotype (STtotal), (2) serotypes included in VCN7 (PCV7), (3) serotypes additional to VCN7 (PCV13-no7), (4) serotypes included in VCN13 (PCV13) and (5) serotypes not included in VCN13 (non-PCV13).

We carried out the following analyses for the five case groups by RAS and penicillin-sensitive serotypes:

1. Evolution of CI, childhood immunisation coverage and DHD. It was analysed by the following:
  1.1. JointPoint models: We estimated the annual trend of vaccine coverage (VC13, VC7 + 13), DHD and cumulative incidence for each of the five groups of IPD. In all cases, we calculated the annual percentage of change (APC) and the average annual percentage of change (AAPC).
  1.2. Poisson models: The annual mean incidence rate ratio (IRR) was estimated for each group of IPD between the pre-vaccination period (2007–2009) and the vaccination period (2011–2016).
2. Evolution of the distribution of individual serotypes:
  2.1. Distribution of individual serotypes: the biannual prevalence of cases of the most frequent serotypes was estimated according to their inclusion in VCN13. Estimates were made using the following four formulas:
    PCV13 specific with RAS-Pen/total PCV13 with RAS-Pen
    Specific sensitive PCV13/total PCV13 sensitive
    Specific non-PCV13 with RAS/total of non-PCV13 with RAS-Pen
    Specific non-PCV13 sensitive/total non-PCV13 sensitive
  2.2. Poisson models: We estimated the annual mean incidence RRs for the most frequent individual serotypes, between the pre-vaccination and the vaccination period. We calculated these for RAS-Pen and penicillin-sensitive serotypes.
3. Association of CI with vaccine coverage and DHD. We analysed these using Poisson multivariate models:
  3.1. Model 1: whose dependent variable was the incidence of IPD (STtotal/PCV13/non-PCV13/PCV13-no7) and the explanatory variables VC13 and the DHD of beta-lactam antibiotics. The analysis period was 2010 to 2016, i.e. since VCN13 was in the childhood immunisation schedule.
  3.2. Model 2: whose dependent variable was the incidence of IPD (PCV7/non-PCV13) and the explanatory variables were VC7 + 13 and HDD. The analysis period was from 2007 to 2016. During this period, VCN7 first and then VCN13 were included in routine vaccination throughout the study period.

The statistical programmes used were JointPoint Trend Analysis 4.5 and STATA v. 14.

## Results

**Fig. 1:**
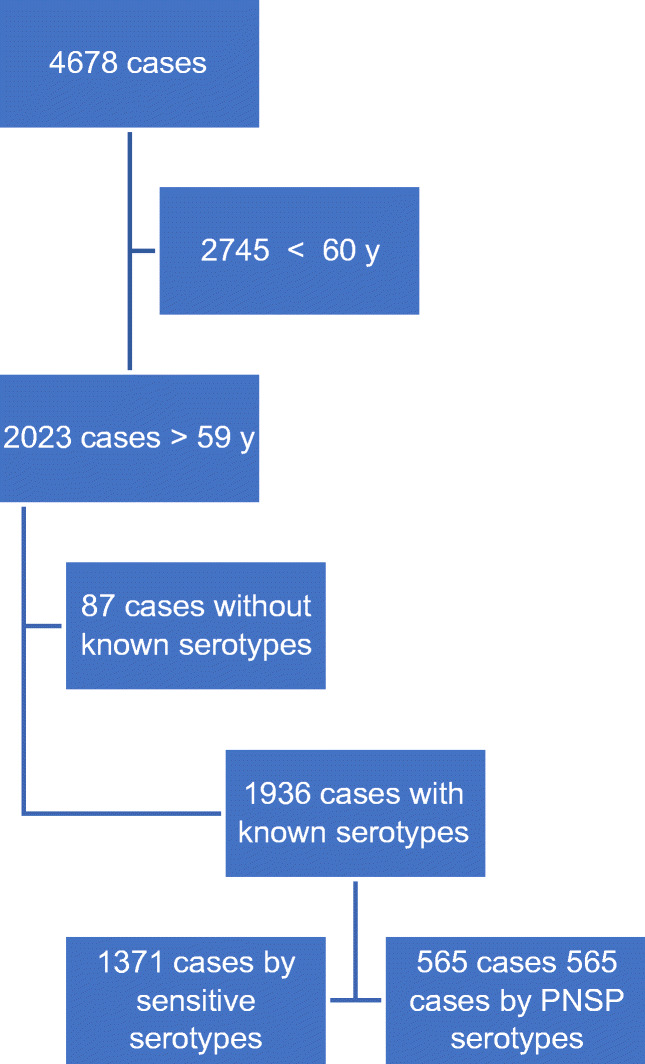
Study population size

There were 4678 cases of IPD reported to the EDO system in the CM, of which 41.4% were cases in adults over 59 years of age with known serotype (1936 cases) (Fig. 1). Among the latter, 565 cases (29.2%) had RAS-Pen serotypes, and the rest were sensitive.

### Evolution of CI, CVC and DHD trends

#### Evolution of CI, CVC and DHD using JointPoint models.

**Fig. 2:**
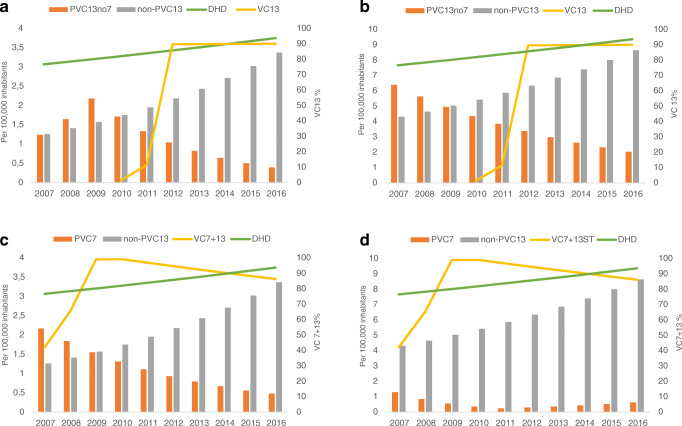
**a-b** Trend in IPD incidence of non-PCV13 and PCV13-no7, DHD and CVC in the Community of Madrid in the 2007–2016 period. **a** Trend in IPD incidence of serotypes with RAS-Pen.**b** Trend in incidence of IPD caused by penicillin-sensitive serotypes. **c–d** Trends in the incidence of IPD by PCV7, non-PCV13, DHD and PCVI7 + 13, in the Community of Madrid in the 2007 to 2016 period

Figure 2a and b show annual trends in CI cases by PCV13-no7 and non-PCV13, CV13 and DHD, obtained with the JointPoint programme. The evolution of the cases was expressed by serotypes with RAS-Pen and susceptible to penicillin separately.

The graphs show that the VC13 has been increasing since its inclusion in 2010, exceeding 90% in 2013, thereafter remaining stable.

The evolution of beta-lactam consumption (DHD) increased throughout the period studied, from 15.64 in 2007 to 17.94 in 2016.

The incidence of PCV13-no7 cases showed a different trend by penicillin sensitivity. Those produced by RAS-Pen serotypes increased until 2009, coinciding with the increase in beta-lactam consumption (DHD). From 2010 they decreased, while the VC13 increased. In contrast, cases of sensitive serotypes tended to decrease throughout the study period.

Non-PCV13 cases tended to increase throughout the study period and were similar among cases of RAS-Pen and sensitive serotypes.

Figure 2c and d show trends in cases of PCV7, compared with the evolution of VC7 + 13, since they were the target serotypes of these vaccines. The trend in the incidence of PCV13 cases decreased for RAS and sensitive pneumococci. The decrease was larger for serotype with RAS. Likewise, VC7 + 13 tended to increase until 2009, with a slight decrease observed until the end of the study period.

Table 1 shows the CMI calculated per 100,000 inhabitants and the analysis of trends obtained using JointPoint. The overall annual CMI trend of cases of IPD declined throughout the study period (AAPC: -1%), with a higher decline for RAS-Pen cases (AAPC: -2.4%) than for SP sensitive cases (AAPC: -0.2%).

**Table 1:** Evolution of vaccination coverage, DHD and CI of cases by serotype group and antibiotic sensitivity. Community of Madrid (2007-2016)

	CMI 100,000 pax		AAPC CI 95%		APC CI 95%			
	2007	2016	2007–2016		First period		Second period	
IPD	15.01	14.1	-1%	(-4.8; 3.1)				
RAS-Pen								
STtotal	4.57	2.64	-2.4%	(-6.7; 2.1)				
PCV13	3.62	0.76	-14.4%*	(-19.4; -9.2)				
Non-PCV13	0.95	1.88	11.5%*	(3; 20.7)				
PCV13no7	1.38	0.39	-12.1%*	(-22.6; -0.1)	2007–2009	32.7% (-31.2; 155)	2009 –2016.	-21.8%* (-29.7; -13)
PCV7	2.24	0.28	-15.5%*	(-23; -7)				
SENSITIVE								
STtotal	10.44	11.46	-0.2%	(-4.6; 4.4)				
PCV13	6.56	2.64	-11.4%*	(-14.5; -8.2)				
Non-PCV13	3.88	8.82	8%*	(2.3; 14.1)				
PCV13no7	5.26	2.01	-12%*	(-15.8; -7.9)				
PCV7	1.29	0.63	-7.7%	(-20.9; 7.7)				
Population variables								
DHD	15.64	17.93	2.3%	(1.3; 3.3)				
VC13	1.34%^	91.69%	98.6%	(56.2; 152.5)	2010–2012	681%* (62.2; 3666)	2012–2016	0.1 (-8.1;9)
VC7 + 13	32.21%	91.69%	8.3%	(4.7;12)	2007–2009	56.7%* (29.5; 89.5)	2009–2016	-2.5%* (-4.2; -0.8)

*If AAPC or APC is *p* < 0.05. ^2010 annual vaccination coverage; *CMI* cumulative incidence, *AAPC* average annual percent change, *APC* annual percent change, *RAS-Pen* with reduced antibiotic sensitivity

Among the five serotype groups studied, we observed a statistically significant decrease in the cases caused by PCV13 and PCV7. The incidence of non-PCV13 cases had an upward trajectory, the trend of cases due to PCV13-no7 with RAS-Pen described two periods, one ascending period followed by a descending period, neither of which was statistically significant. PCV13-no7 sensitive cases did not evolve similarly. Except for cases due to PCV13-no7, the other trends were similar regarding sensitive serotypes and with RAS-Pen, although the differences were greater for serotype with RAS-Pen.

On the other hand, VC13 showed a clear upward trend, much more pronounced in the 2010–2012 period.

Finally, community consumption of beta-lactams increased clearly throughout the period studied.

#### Evolution of CMI of cases by the five groups of serotypes using Poisson models

The average annual CMI of cases in the vaccination period was lower than that in the pre-vaccination period for all cases except for non-PCV13. The change was greater for cases with pneumococci with RAS-Pen (Table 2).

**Table 2:** CMI of IPD by serotypes

RAS-Pen	Serotypes	CMI × 100,000 pax		RR	CI 95%	
		Pre-vaccination	Vaccination period			
YES	STtotal	4.85 (175)	4.08 (335)	0.840	0.698	1.015
	PCV13	3.69 (133)	1.41 (116)	0.382	0.295	0.495
	non-PCV13	1.16 (42)	2.66 (219)	2.289	1.639	3.266
	PCV13no7	1.66 (60)	0.75 (62)	0.454	0.313	0.658
	PCV7	2.02 (73)	0.66 (51)	0.324	0.224	0.468
NO	STtotal	11.64 (420)	10.05 (826)	0.863	0.767	0.973
	PCV13	6.40 (221)	3.12 (256)	0.487	0.406	0.584
	Non-PCV13	5.24 (189)	6.94 (570)	1.324	1.121	1.569
	PCV13no7	5.48 (198)	2.70 (222)	0.492	0.405	0.599
	PCV7	0.915 (33)	0.413 (34)	0.452	0.272	0.753

*RAS-Pen* reduced antibiotic sensitivity to penicillin, *CMI* cumulative incidence, *IRR* incidence rate ratio, *CI* confidence interval. Comparison between periods: pre-vaccination (2007–2009) and vaccination period (2011–2016) until the inclusion of VCN13

### Evolution of differences in individual serotype distribution

#### Distribution of individual serotypes

**Fig. 3:**
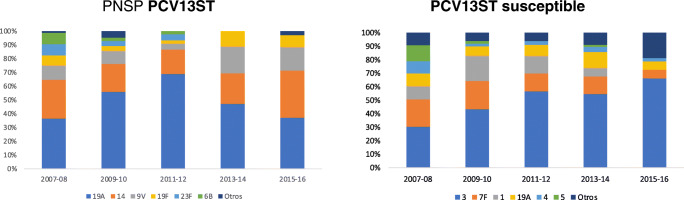
Distribution of serotypes (%) identified in PCV13 pneumococci, by penicillin sensitivity

The cases of PCV13 with RAS-Pen evolved differently from the sensitive cases. The 19A serotype with RAS-Pen decreased the most. The remaining serotypes with RAS decreased in prevalence, except for 14 and 9 V. SP sensitive cases also decreased in prevalence, except for three (Fig. 3).

**Fig. 4:**
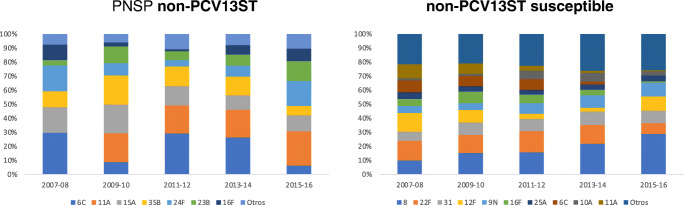
Distribution of serotypes (%) identified in non-PCV13 pneumococci, by penicillin sensitivity

Among the non-PCV13 with RAS-Pen, 6C was prominent at the beginning of the period (5.41%. eight of 148 cases) which then fell to 1.41% (five of 346 cases). Serotype 11A was not identified in the early years, reaching 5.5% at the end of the study (19 cases of 346). Something occurred with the 24F serotype. The frequency of all the other serotypes with RAS-Pen remained stable. In contrast, serotype 8 sensitive cases increased most of all during the study period from 8.11% (12 of 148 cases) to 22.25% (77 of 346 cases) (Fig. 4).

#### Evolution of CMI of individual serotypes using Poisson models (Table 3)

- The inter-period IRR (pre-vaccine/vaccine) for all cases by vaccine serotypes was negative. There was a notable reduction of serotypes 19A and 14 among the cases with RAS-Pen. 7F and 1 were prominent among the sensitive serotypes.
- IRR was positive for cases of non-vaccine serotypes with RAS, with a notable increase in serotypes 6C, 23B and 11A. The increase in the vaccine period of serotype 8 was significant among the sensitive serotypes.

**Table 3:** Evolution of the main serotypes

RAS TO PEN	Serotypes	CMI × 100.000 pax		IRR	CI 95%	
		Pre-vaccine	Vaccination period			
Yes	PCV13					
	19A^1^	1.52 (55)	0.74 (61)	0.487	0.333	0.714
	14	0.99 (36)	0.34 (28)	0.341	0.200	0.575
	9 V	0.36 (13)	0.18 (15)	0.507	0.225	1.157
	19F	0.19 (7)	0.10 (8)	0.502	0.159	1.625
	23F	0.22 (8)	0.02 (2)	0.110	0.011	0.550
	6B	0.25 (9)	0.01 (1)	0.049	0.001	0.352
	non-PCV13					
	6C	0.25 (9)	0.54 (44)	2.146	1.034	5.00
	11A^2^	0.11 (4)	0.57 (47)	5.158	1.886	19.715
	15A	0.25 (9)	0.32 (26)	1.268	0.576	3.076
	35B	0.17 (6)	0.29 (24)	1.756	0.699	5.252
	24F	0.17 (6)	0.28 (23)	1.683	0.666	5.053
	23B	0.08 (3)	0.26 (21)	3.073	0.918	16.091
No	PCV13					
	3^1^	2.27 (82)	1.84 (151)	0.809	0.613	1.071
	7F^1^	1.27 (46)	0.34 (28)	0.267	0.161	0.437
	1^1^	0.78 (28)	0.22 (18)	0.282	0.147	0.528
	19A^1^	0.50 (18)	0.27 (22)	0.537	0.274	1.061
	non-PCV13					
	8^2^	0.67 (24)	1.63 (134)	2.451	1.579	3.959
	22F^2^	0.64 (23)	0.78 (64)	1.222	0.748	2.062
	31	0.44 (16)	0.63 (52)	1.426	0.802	2.677
	12F^2^	0.64 (23)	0.44 (36)	0.687	0.396	1.215
	9N^2^	0.25 (9)	0.62 (51)	2.488	1.213	5.748

^1^PCV13-no7. ^2^Serotypes included in the polysaccharide vaccine (PPSV23) that are not in the VCN13

*CMI* cumulative incidence, *IRR* incidence rate ratio, *CI* confidence interval

Comparison between periods: pre-vaccine (2007–2009) and vaccination period (2011–2016) to the inclusion of VCN13

### Association of CMI with VC13 and DHD.

Table 4 shows the multivariate models developed to analyse the association between case incidence and explanatory variables (CVC and DHD of beta-lactams).

**Table 4:** Association of vaccination coverage and population consumption of beta-lactams with the incidence of IPD by pneumococci according to RAS to penicillin

Models	Variable	RAS-Pen			Sensitive to penicillin		
		RR	CI 95%		RR	CI 95%	
Model 1	STtotal						
	Beta-lactam DHD	1.082	0.894	1.191	1.127*	1.061	1.197
	VC13	0.833*	0.722	0.963	0.912*	0.832	0.999
	PCV13						
	Beta-lactam DHD	0.989	0.842	1.162	0.94	0.843	1.050
	VC13	0.638*	0.501	0.812	0.739*	0.632	0.867
	STnoVCN13						
	Beta-lactam DHD	1.156*	1.025	1.304	1243*	1.154	1.338
	VC13	0.994	0.155	0.828	1038	0.926	1164
	PCV13no7						
	Beta-lactam DHD	0.845	0.67	1.067	0.916	0.814	1.032
	VC13	0.574*	0.413	0.797	0.685*	0.578	0.812
Model 2	STnoVCN13^						
	Beta-lactam DHD	1.281*	1.164	1.408	1.199*	1.137	1.266
	VC7 + 13	1.164	0.975	1.390	0.987	0.898	1.085
	PCV7^						
	Beta-lactam DHD	0.803*	0.691	0.934	0.91	0.748	1.106
	VC7 + 13	0.632*	0.522	0.765	0.647*	0.491	0.851

^The analysis was carried out on incident cases in the 2007–2016 period. *Statistically significant RR

Model 1 shows that CV13 was inversely associated with the incidence of cases by STtotal with RAS-Pen (IRR 0.833) and sensitive (0.832) cases. The association was greater with the cases of PCV13-no7. Community beta-lactam consumption was associated solely with the incidence of non-PCV13 IPD with RAS-Pen (IRR: 1.156) and 2 (1.281).

Model 2 shows that the incidence of cases of PCV7 with RAS-Pen was positively associated with community beta-lactam consumption and negatively associated with VC7 + 13. Those which were penicillin-sensitive were also negatively associated with VC7 + 13.

## Discussion

The indirect effect of childhood vaccine coverage has had an important role in the decrease of RAS-Pen serotype-based IPD in people over 59 years of age [5, 16]. This effect was far greater for cases of vaccine serotypes. In our study, the direct effect of PCV13 vaccine coverage on adults in the CM has not been analysed because the vaccine has not been on the adult immunisation schedule for the entire period studied.

According to our results, the evolution of the incidence of IPD cases in adults over 59 years of age was different for RAS-Pen and sensitivity cases. The evolution of the incidence of the cases by the six serotypes with RAS-Pen additional to 7-valent described two trends. The first of these was ascending until 2010 and then descended until the end of the period studied. The upward trend could be explained by antibiotic pressure, while the downward trend was due to the indirect effect of childhood vaccine coverage. The latter would seem to contradict other studies, such as those by Gossens, which suggest that the higher the use of antibiotics, the higher the incidence of cases by serotypes with RAS-Pen, although it should be noted that these works have not considered the opposite effect of the high childhood immunisation coverage [13]. Unlike the evolution of the six additional serotypes to PCV with RAS-Pen, the sensitive serotypes descended throughout the period studied.

The evolution of the incidence of IPD cases by non-vaccine serotypes was different. At the end of the period studied, their incidence exceeded the level obtained by the vaccine serotype cases. This could be due to the so-called serotype replacement, caused by the increase in cases of non-vaccine serotypes in the vaccinated and unvaccinated population. Replacement was greater among RAS-Pen serotypes than sensitive serotypes, which may be caused not only by the effect of vaccination coverage but also by the constant increase in beta-lactam consumption during the follow-up period.

Studies by Moore in the USA and Andrews in the UK describe the appearance of serotype replacement in the unvaccinated population 3 years after the inclusion of PCV13 in the childhood immunisation schedule [9, 17, 18]. In our study, replacement occurred a little earlier than 3 years after the inclusion of PCV13 (approximately 2012). This could be due to the rapid rise of VC13, which reached 75% in 2012, a value considered by different studies as ideal to trigger a global decline in pharyngeal colonisation by vaccine serotypes and a simultaneous increase in non-vaccine serotypes [19–21].

Regardless of the differences in the evolution of incidences between RAS-Pen and sensitive serotypes, there were also distribution differences. We observed that one of the vaccine serotypes with RAS-Pen, whose incidence decreased the most, was the 19A [22]. This serotype was predominant in Europe in the period before the inclusion of PCV13, except for Finland, where it was serotype 14.

Among the cases of vaccine sensitive serotypes, it was also those additional to the 7-valent vaccine that decreased most after the year 2010, except serotype 3, which was the only one that increased during the entire period studied. This could be because it is a serotype that does not colonise the nasopharynx in children. Because of this, the indirect vaccine effect would be very limited in adults [23]. Direct vaccination would be the most effective way to eradicate pharyngeal colonisation of serotype 3 in adults.

Among the non-vaccine serotypes with RAS-Pen whose incidence increased at the end of the studied period are 11A, 6C, 35B and 23B. In other European studies such as the one by van der Linden in Germany, 15A and 23B were the most frequent [8]. Outside Europe, the most frequent serotypes in the USA were 15B, 23A, 23B and 35B [24]. These data show the significant difference between countries in the distribution of emerging serotypes, although the results of the above studies are not specific to the cohort of adults over 59 years of age.

Non-vaccine sensitive serotypes also became more prevalent, the most prominent being 8, although the magnitude of the increase in incidence and prevalence of cases due to sensitive serotypes was lower than that presented by serotype with RAS-Pen.

We do not yet know the predisposition of some serotypes to acquire antibiotic resistance. Among these are 19A (PCV13) and 11A (non-PCV13) serotypes that were frequent and predominant among the serotypes with RAS-Pen. However, there are other serotypes which, although frequent, like the above, are almost always sensitive, such as serotypes 3 (PCV13) and 8 (non-PCV13). We know that PS acquires resistance from genes from other PS and from commensal *Streptococcus* in a process that could be favoured by the increase in antibiotic pressure and the immunological selection of vaccines.

The different predisposition of serotypes could likely be explained by microbiological factors, including those concerning fitness and the cost associated with resistance. However, some clones of serotypes with RAS-Pen could evade the host’s immune response by adopting a non-vaccine serotype capsule. One such genotypic clones that would facilitate this camouflage is ST156, associated with serotypes 9 V and 14, in the period before the inclusion of PCV13 [25] that is currently associated with serotypes not included in PCV13. One of the latter would be 11A, which successfully avoids the immune response of the host. Such circumstances make 11A an emerging serotype. Another non-vaccine serotype whose incidence is increasing in some countries in our environment is 7C. In this case, this is due to the expansion of clone 177, which was previously associated with the 19F vaccine serotype [26].

The main strength of this study is that the variables come from the Mandatory Notification System for Infectious Diseases System of the Community of Madrid (EDO), which gathers population data actively and consistently and did not undergo any relevant changes during the 2007–2016 period. Another strength is the work done by microbiology laboratories managing samples for serotyping since the serotype was only unknown in 5% of cases.

A weakness of this study is a lack of data for determining whether the changes detected are due to other factors such as microbiology, secular disease trend, changes in reporting, chance or the presence of other factors such as socio-economic precariousness that could play an important role in the evolution of the IPD by serotypes with RAS [2]. Nor did we consider the intervention of the coverage of the polysaccharide vaccine 23 (PPSV23), partly because the scientific literature does not give it a relevant role in the epidemiology of resistant serotypes [27]. Unlike conjugate vaccines, PPSV23 does not generate lymphocyte-related immune memory, nor does it intervene in pharyngeal colonisation of SP, both important processes that explain the transmission and dissemination of antibiotic-resistant serotypes [28, 29]. This is demonstrated by an increase rather than a decrease in the incidence of serotypes with RAS that are included in PPSV23 but not in PNV13 (Table 3). Thus, the evolution of these serotypes of PPSV23 is shown in that of serotypes not included in PCV13.

We can conclude that our study demonstrates a reduction in the incidence of IPD produced by serotypes with RAS-Pen in adults over 59 years of age, at the expense of vaccine serotypes, which supports the existence of vaccine herd immunity. In contrast, the incidence of non-vaccine serotypes with RAS-Pen increased more than that of sensitive serotypes, possibly due to the continued increase in beta-lactam consumption. The distribution of non-vaccine serotypes was different according to their sensitivity to penicillin. Non-vaccine serotypes with RAS-Pen include 6C, 11A and 23B, and among the sensitive ones, 8.

The results of this work provide the basis for guiding vaccination policies and rational use of antibiotics. In the future, research into vaccines with new serotypes could be valuable to public health interventions to control RAS-Pen serotype-induced IPD. In any case, it is essential to continue with active, epidemiological and microbiological surveillance programmes to assess the effect of vaccination on the incidence of invasive disease [30, 31].

## Data Availability

Yes. The datasets generated during and/or analysed during the current study are available from the corresponding author on reasonable request.

## References

[1] Liñares J, Ardanuy C, Pallares R, Fenoll A (2010). Changes in antimicrobial resistance, serotypes and genotypes in Streptococcus pneumoniae over a 30-year period. Clin Microbiol Infect Off Publ Eur Soc Clin Microbiol Infect Dis..

[2] Lynch JP, Zhanel GG (2010). Streptococcus pneumoniae: epidemiology and risk factors, evolution of antimicrobial resistance, and impact of vaccines. Curr Opin Pulm Med..

[3] 23-valent pneumococcal polysaccharide vaccine (2008). WHO position paper. Releve Epidemiol Hebd..

[4] Shea KM, Edelsberg J, Weycker D, Farkouh RA, Strutton DR, Pelton SI. Rates of pneumococcal disease in adults with chronic medical conditions. Open Forum Infect Dis. 2014 [cited 2018 Oct 23];1(1). Available from: https://academic.oup.com/ofid/article/1/1/ofu024/2280681 10.1093/ofid/ofu024 PMC4324183 25734097

[5] The worldwide impact of the seven-valent pneumococcal... : the pediatric infectious disease journal. LWW. [cited 2017 Jul 13]. Available from: http://journals.lww.com/pidj/Fulltext/2012/05000/The_Worldwide_Impact_of_the_Seven_valent.16.aspx 10.1097/INF.0b013e31824de9f6 22327872

[6] Hays C, Vermee Q, Agathine A, Dupuis A, Varon E, Poyart C (2017). Demonstration of the herd effect in adults after the implementation of pneumococcal vaccination with PCV13 in children. Eur J Clin Microbiol Infect Dis Off Publ Eur Soc Clin Microbiol..

[7] Bonten MJM, Huijts SM, Bolkenbaas M, Webber C, Patterson S, Gault S (2015). Polysaccharide conjugate vaccine against pneumococcal pneumonia in adults. N Engl J Med..

[8] van der Linden M, Perniciaro S, Imöhl M (2015). Increase of serotypes 15A and 23B in IPD in Germany in the PCV13 vaccination era. BMC Infect Dis..

[9] Tin Tin Htar M, Christopoulou D, Schmitt H-J (2015). Pneumococcal serotype evolution in Western Europe. BMC Infect Dis.

[10] Moreno-Pérez D, Álvarez García FJ, Arístegui Fernández J, Barrio Corrales F, Cilleruelo Ortega MJ, Corretger Rauet JM (2012). Calendario de vacunaciones de la Asociación Española de Pediatría: recomendaciones 2012. An Pediatría.

[11] Hicks LA, Chien Y-W, Taylor TH, Haber M, Klugman KP, on behalf of the Active Bacterial Core Surveillance (ABCs) Team (2011). Outpatient antibiotic prescribing and nonsusceptible Streptococcus pneumoniae in the United States, 1996-2003. Clin Infect Dis..

[12] Granizo JJ, Aguilar L, Casal J, García-Rey C, Dal-Ré R, Baquero F (2000). Streptococcus pneumoniae resistance to erythromycin and penicillin in relation to macrolide and β-lactam consumption in Spain (1979–1997). J Antimicrob Chemother..

[13] Goossens H, Ferech M, Vander Stichele R, Elseviers M (2005). Outpatient antibiotic use in Europe and association with resistance: a cross-national database study. The Lancet..

[14] Richter SS, Heilmann KP, Dohrn CL, Riahi F, Beekmann SE, Doern GV (2009). Changing epidemiology of antimicrobial-resistant Streptococcus pneumoniae in the United States, 2004–2005. Clin Infect Dis..

[15] WHO | The anatomical therapeutic chemical classification system with defined daily doses (ATC/DDD). WHO. [cited 2018 Oct 22]. Available from: http://www.who.int/classifications/atcddd/en/

[16] Predictive value of pneumococcal nasopharyngeal cultures... : the pediatric infectious disease journal. LWW. [cited 2017 Jul 13]. Available from: http://journals.lww.com/pidj/Fulltext/2000/04000/Predictive_value_of_pneumococcal_nasopharyngeal.7.aspx

[17] Moore MR, Link-Gelles R, Schaffner W, Lynfield R, Lexau C, Bennett NM (2015). Effect of use of 13-valent pneumococcal conjugate vaccine in children on invasive pneumococcal disease in children and adults in the USA: analysis of multisite, population-based surveillance. Lancet Infect Dis..

[18] Andrews NJ, Waight PA, Burbidge P, Pearce E, Roalfe L, Zancolli M (2014). Serotype-specific effectiveness and correlates of protection for the 13-valent pneumococcal conjugate vaccine: a postlicensure indirect cohort study. Lancet Infect Dis..

[19] Lindstrand A, Galanis I, Darenberg J, Morfeldt E, Naucler P, Blennow M (2016). Unaltered pneumococcal carriage prevalence due to expansion of non-vaccine types of low invasive potential 8 years after vaccine introduction in Stockholm, Sweden. Vaccine..

[20] Muñoz-Almagro C, Jordan I, Gene A, Latorre C, Garcia-Garcia JJ, Pallares R (2008). Emergence of invasive pneumococcal disease caused by nonvaccine serotypes in the era of 7-valent conjugate vaccine. Clin Infect Dis..

[21] Loughlin AM, Hsu K, Silverio AL, Marchant CD, Pelton SI (2014). Direct and indirect effects of PCV13 on nasopharyngeal carriage of PCV13 unique pneumococcal serotypes in Massachusetts’ children. Pediatr Infect Dis J..

[22] Serotype 19A Is the most common serotype causing invasive pneumococcal infections in children | Articles | Pediatrics. [cited 2017 Dec 16]. Available from: http://pediatrics.aappublications.org/content/125/3/429?pagewanted=all 10.1542/peds.2008-1702 20176669

[23] Moore MR, Link-Gelles R, Schaffner W, Lynfield R, Holtzman C, Harrison LH (2016). Effectiveness of 13-valent pneumococcal conjugate vaccine for prevention of invasive pneumococcal disease in children in the USA: a matched case-control study. Lancet Respir Med..

[24] Richter SS, Diekema DJ, Heilmann KP, Dohrn CL, Riahi F, Doern GV (2014) Changes in pneumococcal serotypes and antimicrobial resistance after introduction of the 13-valent conjugate vaccine in the United States. Antimicrob Agents Chemother. AAC.03344-14 10.1128/AAC.03344-14 PMC4249410 25136018

[25] Aguinagalde L, Corsini B, Domenech A, Domenech M, Cámara J, Ardanuy C (2015). Emergence of amoxicillin-resistant variants of Spain9 V-ST156 pneumococci expressing serotype 11A correlates with their ability to evade the host immune response. PloS One..

[26] Makwana A, Ladhani SN, Kapatai G, Campion E, Fry NK, Sheppard C (2018). Rapid spread of pneumococcal nonvaccine serotype 7C previously associated with vaccine serotype 19F, England and Wales. Emerg Infect Dis..

[27] Mäkelä PH, Käyhty H (2002). Evolution of conjugate vaccines. Expert Rev Vaccines..

[28] Poolman J, Borrow R (2011). Hyporesponsiveness and its clinical implications after vaccination with polysaccharide or glycoconjugate vaccines. Expert Rev Vaccines..

[29] Russell FM, Carapetis JR, Balloch A, Licciardi PV, Jenney AWJ, Tikoduadua L (2010). Hyporesponsiveness to re-challenge dose following pneumococcal polysaccharide vaccine at 12 months of age, a randomized controlled trial. Vaccine..

[30] Rodríguez MAG, González AV, Gavín MAO, Martínez FM, Marín NG, Blázquez BR (2011). Invasive pneumococcal disease: association between serotype, clinical presentation and lethality. Vaccine..

[31] Moraga-Llop FA (2009). Pneumococcal disease and emergence of serotypes in the vaccine era. Transient trends and serotype replacement?. Enferm Infecc Microbiol Clin..

